# Roles of ER Membrane Protein Complex in Protein Biogenesis and Quality Control in the Lung and Beyond

**DOI:** 10.1111/cpr.70191

**Published:** 2026-03-09

**Authors:** Yan Qiao, Yuqing Sun, Yutao Huang, Qinghang Meng, Xinhua Lin, Wei Wei, Xiaofang Tang

**Affiliations:** ^1^ School of Life Sciences, Inner Mongolia University Hohhot China; ^2^ Greater Bay Area Institute of Precision Medicine (Guangzhou) Guangzhou China; ^3^ School of Life Sciences, Fudan University Shanghai China; ^4^ Department of Respiratory and Critical Care Medicine State Key Laboratory of Respiratory Health and Multimorbidity, West China Hospital, Sichuan University Chengdu Sichuan China

**Keywords:** EMC, lung model, protein biogenesis, protein homeostasis, protein quality control

## Abstract

The endoplasmic reticulum membrane protein complex (EMC) is an evolutionarily conserved multi‐subunit complex. Due to its essential roles in protein biogenesis and quality control, the EMC has attracted considerable attention in recent years. In this review, we systematically explore the functions and disease‐associated regulatory mechanisms of the EMC across various organ systems. We highlight the lung as a paradigmatic model for illustrating the ‘molecular switch’ function of EMC shaped by spatiotemporal and cell‐type‐specific contexts. Dysfunction of EMC contributes to pathologies and cancers of diverse organs, positioning EMC subunits as potential biomarkers and therapeutic targets. Despite considerable progress, our understanding of the molecular underpinnings of EMC in health and disease remains far from complete. Future efforts should aim to unravel the regulatory networks centered on EMC to harness their potential for cross‐disease therapy development.

## Graphical abstract

The endoplasmic reticulum membrane protein complex (EMC) is an evolutionarily conserved, multi‐subunit transmembrane protein complex crucial to membrane protein biogenesis and cellular protein quality control. This review systematically examines the structure, functions and disease‐associated regulatory mechanisms of EMC across multiple organ systems, with particular emphasis on pulmonary pathophysiology.

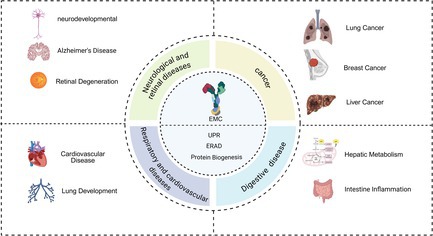

## 1 Introduction

Endoplasmic Reticulum (ER) is often described as a maze‐like structure present in the eukaryotic cell with smooth membrane vesicles. ER serves as the central biosynthetic organelle for secretory and membrane proteins [1]. Through coordinating co‐translational translocation, post‐translational modifications and folding, the ER achieves functional protein conformations. Critically, it also keeps strict quality control by misfolded proteins recognition and degradation guidance, thereby preventing toxic accumulation [2, 3, 4, 5, 6].

In recent decades, EMC has emerged as a pivotal regulator among the diverse molecular machineries governing ER functions. EMC is essential for cellular homeostasis maintenance. This is underscored by the broad spectrum of developmental and pathological disorders related to EMC's dysfunction, including neurodegenerative diseases, inherited retinal dystrophies [7] and cardiopulmonary disorders [8, 9, 10, 11]. Table 1 summarises the disease associations of specific EMC subunits.

**TABLE 1:** Reported function of EMC subunits and their involvement in diseases.

Related disease	Main model	Molecular mechanism	Main outcome	Types of clinical samples
Global developmental delay, hypotonia, scoliosis and cerebellar atrophy	Human	Biallelic or monoallelic *EMC1* variants might cause perturbations of protein folding and organelle crosstalk and thus lead to a syndrome including intellectual disability and preferential degeneration of the cerebellum [12].	Homozygous variants in EMC1 segregated with a phenotype of developmental delay, hypotonia, scoliosis and cerebellar atrophy in three families.	Individuals with Global Developmental Delay, Hypotonia, scoliosis and Cerebellar Atrophy
Defective locomotion	*Drosophila*	*Emc1* depletion could increase the transcript levels of *Ire1*, *PERK* and *Atf6* [13].	Emc1 is required for the sarcoplasmic reticulum and mitochondrial functions in the *Drosophila* muscle.	Non‐clinical validation
Neural crest defects	*Xenopus laevis*	Loss of *Emc1* reduced WNT signalling and Frizzled (Fzd) levels in neural crest cells [14].	Disrupted EMC‐mediated topogenesis drives congenital neural crest defects.	hESC
Familial exudative vitreoretinopathy (FEVR)	Mouse	Loss of *Emc1* led to compromised beta‐catenin signalling activity through reduced expression of Wnt receptor FZD4 [15].	Loss of *Emc1* in endothelial cells led to reduced vascular progression and vascular density, diminished tip cell sprouts and vascular leakage	FEVR patients
Inherited retinal diseases (IRDs)	Zebrafish	Knockdown of *Emc1* results in ER stress and activates UPR in zebrafish eyes [7].	Emc1 is essential for vision and zebrafish photoreceptor outer segment morphogenesis.	Non‐clinical validation
Red‐light blindness	Zebrafish	The zebrafish Emc3, Pob, is not involved in phototransduction but rather plays an essential role in protein sorting and/or trafficking [16].	Pod mutant larvae showed defective optokinetic responses in red but not white light.	Non‐clinical validation
Retinal morphological degeneration	Mouse	Loss of *Emc3* induces UPR in retinal cells, ultimately driving apoptosis through sustained ER stress [17].	EMC3 is essential for retinal organisation and neurogenesis during mouse retinal development.	Non‐clinical validation
Retinitis pigmentosa	Mouse/*Drosophila*	ER complex proteins are required for rhodopsin biosynthesis and photoreceptor survival in *Drosophila* and mice [18].	EMC3 is important for M‐opsin stability	Non‐clinical validation
Retinal degeneration	*Drosophila*	EMC is a key factor in the biogenesis of multi‐pass transmembrane proteins, including Rh1 [19].	dPob/EMC3 deficiency induces rhabdomere degeneration in a light‐independent manner.	Non‐clinical validation
Retinitis pigmentosa	Mouse	EMC6 deficiency resulted in diminished expression levels of cilia‐associated proteins ANO2 and TMEM67.	The endoplasmic reticulum membrane protein complex subunit *Emc6* is essential for rhodopsin localization and photoreceptor cell survival [20].	Non‐clinical validation
Defective rhodopsin homeostasis	Mouse	*Emc3*, *Emc5* and *Emc6* mutations decreased Rh1 protein levels and impaired phototransduction, despite proper rhodopsin folding and absence of ERAD‐mediated degradation in these mutants [18].	ER complex proteins are required for rhodopsin biosynthesis and photoreceptor survival in *Drosophila* and mice.	Non‐clinical validation
Interstitial lung disease (ILD)	Mouse/Human	EMC3 and VCP could modulate SP‐C(I73T) transport through coordinated ER‐endocytic machinery crosstalk in the lung epithelia [21].	EMC3 regulates trafficking and pulmonary toxicity of the *SFTPC* ^*I73T*^ mutation associated with interstitial lung disease.	S‐PC(I73T)‐expressing iAT2 cells derived from S‐PC (I73T) patient‐specific iPSCs
Neonatal respiratory failure	Mouse	EMC3 deficiency caused misprocessing and accumulation of surfactant proteins, probably inducing ER stress [8].	EMC3 coordinates surfactant protein and lipid homeostasis required for respiration.	Non‐clinical validation
	Mouse	EMC3 interacts with VCP to regulate the levels and activity of Aurora A in mesenchymal cells [22].	EMC3 deficiency causes spindle assembly defects, cell cycle arrest and apoptosis.	HeLa cell line
Gut Microbial Dysbiosis	Mouse	EMC3 deficiency leads to ER stress [23].	EMC3 maintains intestinal homeostasis by preserving secretory lineages.	Non‐clinical validation
Intestinal epithelium homeostasis imbalance	Mouse	EMC3 is important to the biogenesis of the cystic fibrosis transmembrane conductance regulator (CFTR) maintaining intracellular calcium homeostasis [24].	EMC3 is critical for CFTR function and calcium mobilisation in the mouse intestinal epithelium.	Non‐clinical validation
	*Saccharomyces cerevisiae*	Emc4, by its chaperone activity, folds and stabilises the destabilised and unfolded eIF2Bβ and eIF2Bγ subunits [25].	Emc4 plays a crucial role in eIF2B‐mediated translation regulation and survival under stress conditions.	Non‐clinical validation

*Note*: This table focuses on non‐cancer diseases associated with EMC mutations.

Cellular proteostasis is fundamental to the development and functional integrity of diverse tissues and organs. The lung serves as a prime example of this dependency. Trafficking and quality control of proteins are critical for alveolar formation, surfactant production and maintenance of respiratory function [26, 27]. Despite the growing recognition of EMC's importance, its tissue‐specific regulatory networks, particularly in the lung and its precise molecular mechanisms in disease pathogenesis remain incompletely characterised and lack systematic elucidation. To address this gap, we employ the lung as a model system to decipher the pathophysiology of the EMC. This rationale stems from the pivotal role of alveolar type II (AT2) cells, which synthesise pulmonary surfactant. The metabolic process of surfactants highly depends on the accurate expression, folding and transport of transmembrane proteins, notably ABCA3 and surfactant protein C (SP‐C) [21, 28, 29], in which EMC is directly implicated. Consequently, the lung represents a sensitive physiological system where EMC disruption can manifest clinically, ranging from neonatal respiratory distress syndrome to interstitial lung disease (ILD) [8, 21].

Centering on EMC dysfunction in pulmonary surfactant homeostasis within AT2 cells, this review interrogates how the lung model unveils fundamental principles. These include the EMC's role as a proteostatic ‘molecular switch’, its spatiotemporal regulation manifest in divergent functions across alveolar development, and the mechanistic conservation of diseases in the brain, heart and beyond. By synthesising these insights across organ systems, we aim to move beyond siloed perspectives, arguing for a unified theoretical foundation that informs cross‐system therapeutic strategies and provide a theoretical foundation for cross‐system therapeutic strategies.

## 2 Structure and Evolution of the EMC

Elucidating the precise functions of EMC is fundamentally reliant on understanding its structural organisation. Initially identified in *Saccharomyces cerevisiae* as a conserved six‐subunit transmembrane complex essential for ER protein folding [1], EMC was later defined in humans as a fully assembled ten‐subunit complex (EMC1‐EMC10) through high‐resolution cryo‐electron microscopy [30]. Distinct from other ER‐resident factors involved in membrane protein biogenesis, EMC exhibits a unique architecture characterised by a transmembrane domain (TMD), serving as a central integrating extensive cytoplasmic and luminal regions [1, 31, 32]. This architectural organisation provides a structural blueprint for the multifunctional properties of the EMC, prompting a compelling evolutionary question: How did such a sophisticated macromolecular machine evolve from simpler progenitors?

A key finding in tracing the EMC's evolutionary origins is that EMC3, a core subunit of the transmembrane module, belongs to the conserved Oxa1/Alb3/YidC protein family [33, 34], which is widely distributed in both prokaryotes and eukaryotes. Among these, the prokaryotic YidC protein is the structurally simplest prototype and serves as a foundational model for deciphering the mechanisms and evolutionary trajectory of EMC. Comprehending these evolutionary aspects enhances our understanding of EMC's significance in cell proliferation and protein biogenesis.

In *Escherichia coli* , YidC comprises six transmembrane helices that assemble into a compact globular structure within the membrane [35]. A defining feature is a hydrophilic groove formed by the transmembrane segments TM2, TM3, TM4 and TM6 [36]. The hydrophilicity of the client groove structures is important for substrate binding [37]. This captures the substrate TMD from the lateral gate of the SecYEG translocon, orienting the polar domains of substrates toward the cytosol to ensure their correct integration into the lipid bilayer [35, 38]. This groove‐mediated mechanism is functionally conserved in the eukaryotic EMC. Furthermore, YidC features a large periplasmic domain located between TM1 and TM2. TM1 of YidC functions as an uncleaved signal sequence and is important for membrane insertion of YidC, but not for YidC activity [39, 40].

**FIGURE 1:**
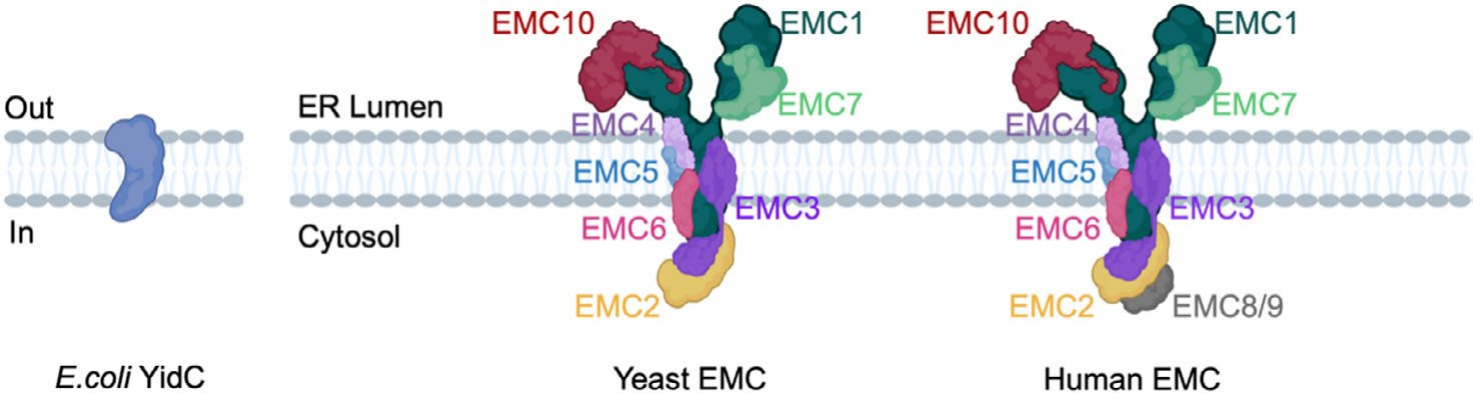
Structure of the EMC. The composition and structure of EMC in yeast and human are highly conserved, except for the absence of two soluble subunits, EMC8/9 in yeast. The eukaryotic EMC transmembrane region contains a large cavity surrounded by EMC3, EMC4 and EMC6, which is proposed to be a TMD‐binding pocket to facilitate insertion of a client TMD into the ER membrane. This structure highly resembles the client‐binding groove found in the prokaryotic insertase YidC.

Evolutionary progression from a dedicated insertase to a multifunctional chaperone complex is marked by significant structural elaboration. In yeast, EMC has evolved to incorporate a luminal cap formed by the subunits Emc1, Emc7 and Emc10, and a C‐terminal loop of Emc4. The cytosolic region of the yeast EMC is composed of Emc2 and the cytosolic domain of Emc3, a N‐terminal loop of Emc4 and a C‐terminal loop of Emc5 [1, 41]. Emc2 forms a shallow, such architecture allows a large substrate to access the cavity in the transmembrane region from the cytosol (Figure 1). This represents an evolutionary transition from a dedicated insertase (YidC) to a functionally specialised complex (yeast EMC), culminating in a multifunctional machine that integrates insertion, folding and quality control (human EMC). Structurally, although the yeast and human EMC share compositional similarities, they display significant conformational differences [1]. In mammals, EMC further evolved into a highly integrated ten‐subunit molecular machine. While sharing core components with yeast, the mammalian EMC incorporates additional soluble subunits, EMC8 and EMC9, which are absent in yeast and are crucial for maintaining complex stability and enabling handling of a broader repertoire of substrate TMDs [42].

Structural analyses indicate that the mammalian EMC consists of ten subunits, categorised into three soluble subunits (EMC2, EMC7 and EMC10) and five transmembrane subunits (EMC1, EMC3–EMC6) [43]. Collectively, these subunits assemble into a compact cluster comprising 12 TMDs. Of these, EMC2, EMC8 and EMC9 are localised to the cytoplasm, while the remaining subunits serve as integral membrane proteins, with each containing one to three TMDs [34]. High‐resolution structural studies further reveal that Client TMDs first enter the cytosolic vestibule formed by either the EMC2/EMC8 or EMC2/EMC9 complex. This vestibule leads into an intramembrane groove that is laterally open toward the lipid bilayer [42]. EMC3, EMC4 and EMC6 form a TMD‐binding pocket (also known as a gated cavity) [1, 41, 44] that stabilises the hydrophobic TMDs of client proteins prior to their integration into the lipid bilayer, thereby supporting the core insertase activity of EMC. The gated cavity is hydrophilic on the cytoplasmic side and becomes increasingly hydrophobic toward the luminal side [1]. EMC1, EMC7 and EMC10 form a luminal domain situated above the transmembrane core [44]. Mutations in this luminal domain have been linked to loss of the EMC complex [45], a trafficking delay for membrane protein Pma1 [46] and male infertility [47]. Additionally, EMC possesses a second membrane‐accessible cavity, which is filled with lipids and bordered by EMC1, EMC3, EMC5 and EMC6 [1]. This second cavity is essential for both insertase‐dependent and insertase‐independent functions of EMC.

Mechanistically, EMC facilitates energetically efficient membrane protein insertion via two key adaptations. First, it induces a local membrane thinning by approximately 10 Å, effectively reducing the distance that a protein's luminal domain must traverse through the hydrophobic lipid environment [48, 49, 50]. Second, EMC strategically positions polar and charged residues within the transmembrane region, which likely serves as a docking site for luminal segments and helps enforce the positive‐inside rule, thereby stabilising the hydrophilic residues often enriched in TMDs of EMC substrates [29, 51].

This evolutionarily refined architecture solidifies EMC's role as a central regulator of ER membrane protein biogenesis and quality control. While the conserved transmembrane core underscores its ancestral insertase function, the acquisition of modular luminal and cytoplasmic elements correlates with expanded client specificity and integrated capabilities of proteostasis—features highly relevant to tissue‐specific functions. However, the absence of high‐resolution structures of EMC bound to disease‐relevant client proteins currently limits a mechanistic understanding of substrate recognition and processing failures in pathology.

## 3 The Lung as a Paradigm: Unveiling the Core Mechanisms of EMC Function

EMC possesses a distinctive architecture, characterised by a large hydrophilic intramembrane cavity that confers highly specific client recognition. To dissect how this molecular machinery coordinates membrane protein biogenesis with quality control, we now leverage the lung—and specifically, AT2 cells—as a physiological model. The high demand in these cells for the synthesis and assembly of surfactant proteins and lipids provides an ideal context to elucidate the core mechanistic principles of EMC function.

### 3.1 EMC in the Biogenesis of Membrane Proteins

The unique architecture of EMC is specialised for a central biological function: ensuring the efficient and accurate biogenesis of membrane proteins [50]. The EMC does not indiscriminately act on all membrane proteins; instead, its substrate selectivity is primarily determined by the physicochemical properties of the client TMDs. A key feature of EMC‐dependent substrates is the possession of TMDs with low hydrophobicity, which are poorly recognised by the canonical Sec61 translocon [52, 53]. This principle is clearly demonstrated in AT2 cells. Our research previously demonstrated that EMC3 regulates the biogenesis of the phospholipid transporter ABCA3 [8], emphasising that the stability of multi‐pass transmembrane proteins is critically dependent on the hydrophilic pocket formed by EMC3, EMC4 and EMC6. EMC3 is positioned to capture substrates for insertion through the hydrophilic vestibule along the surface of EMC3 and EMC6. The EMC decreases the energetic barrier for insertion via local thinning of the membrane and a positively charged patch in the bilayer. The structural basis for this selectivity is attributed to the cavity's capacity to provide a stabilising environment for marginally hydrophobic TMDs during their insertion [50]. The flexible TMDs of EMC4, EMC7 and EMC10, which form a lateral gate of the substrate‐binding groove, facilitate the bilayer sampling by the substrate TMD [50, 54]. However, current research faces significant limitations. Most importantly, the specific TMDs within ABCA3 that directly interact with the EMC pocket, as well as the detailed nature of their interactions, remain speculative due to the absence of high‐resolution complex structures.

The principle demonstrated by the lung model is consistently supported across various cellular systems. As summarised in Table 1, loss of EMC5 or EMC6 specifically disrupts the ER insertion of squalene synthase (SQS) in U2OS osteosarcoma cells, resulting in its mislocalization and aggregation. This phenotype aligns with the function of the EMC5–EMC6 subdomain in forming a conserved insertion channel [28]. Similarly, EMC deficiency leads to the mislocalization of other client proteins, including specific GPCRs and the plasma membrane protein Mrh1‐GFP [45]. Notably, EMC exhibits distinct substrate selectivity toward TMDs with low hydrophobicity, particularly those atypical TMDs in GPCRs that are poorly recognised by the canonical SEC61 translocon [55]. This functional division of labour is further illustrated in the biogenesis of the β1‐adrenergic receptor (β1AR): reconstitution studies indicate that EMC is specifically required for the co‐translational insertion of the first TMD, whereas SEC61 is essential for subsequent TMDs. In the absence of EMC, TMD1 is frequently inserted in an inverted orientation or fails to integrate altogether [56].

How does EMC support the biogenesis of membrane proteins? Mechanistically, EMC can mediate membrane protein insertion either co‐translationally and or post‐translationally. While tail‐anchored (TA) membrane proteins with high hydrophobicity TMDs are targeted by GET3 to the GET1–GET2 complex for membrane insertion [13, 57], EMC serves as an alternative TMD insertase for TA protein substrates with low or moderate hydrophobicity, probably through targeting by calmodulin (CaM) or SGTA (small glutamine‐rich tetratricopeptide repeat‐containing protein alpha) [28]. EMC is also involved in the biogenesis of multipass transmembrane proteins by co‐translationally interacting with substrates through two potential pathways: either by collaborating with specialised membrane chaperones (e.g., Sop4 and Gsf2), or by interacting directly with multipass transmembrane client proteins early during their synthesis, insertion or folding, independent of and possibly prior to chaperone engagement [29]. Functionally, EMC integrates insertase activity with TMD chaperone capability and interfaces with downstream ER processes such as protein degradation and maturation. This multifunctional profile supports an evolutionary model in which an ancient insertase module [33] acquired peripheral subunits that conferred additional chaperone and triage activities. Such complexity explains the broad range of substrates and biological processes affected by EMC deficiency.

### 3.2 The EMC Complex as a Molecular Switch in Protein Quality Control

The biological function of EMC extends beyond acting as a simple membrane protein insertase [52]; it serves as a central regulatory hub within the ER protein quality control network, playing a critical role in maintaining cellular protein homeostasis through a ‘molecular switch’ mechanism [58, 59]. This switch mediates bidirectional regulation. It actively promotes the correct folding of client proteins to support cellular health (the ‘on’ state); conversely, when its own function is compromised or when misfolded proteins accumulate beyond cellular tolerance levels, EMC transitions to a degradation and stress compensation mode (the ‘off’ state). Importantly, EMC directly integrates two core PQC pathways: the unfolded protein response (UPR), ER‐associated degradation (ERAD), highlighting this mechanism's prominence in lung tissue [60, 61].

This continuous requirement for accurate membrane protein insertion and folding renders AT2 cells particularly sensitive to perturbations in EMC activity. During lung development, the high secretory and biosynthetic demands of AT2 cells [62, 63, 64] create a physiologically relevant setting in which the bidirectional regulation of EMC ‘molecular switch’ can be directly interrogated. The core mechanisms governing EMC‐mediated protein quality control are tightly coupled to the fundamental requirements of establishing respiratory function.

#### 3.2.1 The ‘ON’ State: Folding Assistance Under Homeostasis

During the late embryonic to neonatal period, AT2 cells undergo a marked increase in surfactant synthesis and secretion [65]. This process critically relies on the accurate folding and membrane integration of highly hydrophobic proteins, including S‐PC and ABCA3 [8], creating a stringent reliance on the ‘on’ state function of the EMC. At this stage, client TMDs first enter the cytosolic vestibule formed by either the EMC2/EMC8 or EMC2/EMC9 complex. EMC functions as the primary executor of the ‘switch’, with its transmembrane core module (EMC3/6) directly engaging the substrate's TMD via a hydrophilic cavity‐hydrophobic vestibule architecture [50], facilitating efficient folding and integration.

Collectively, these activities ensure efficient surfactant synthesis and secretion, maintain ER proteostasis and are critical for neonatal respiratory function. At a broader level, elucidating the mechanistic principles of EMC‐mediated folding in AT2 cells provides a framework for understanding how multi‐subunit ER membrane complexes coordinate protein biogenesis and quality control in highly secretory tissues.

#### 3.2.2 Stress Compensation and Degradation Activation During Protein Folding Crises

**FIGURE 2:**
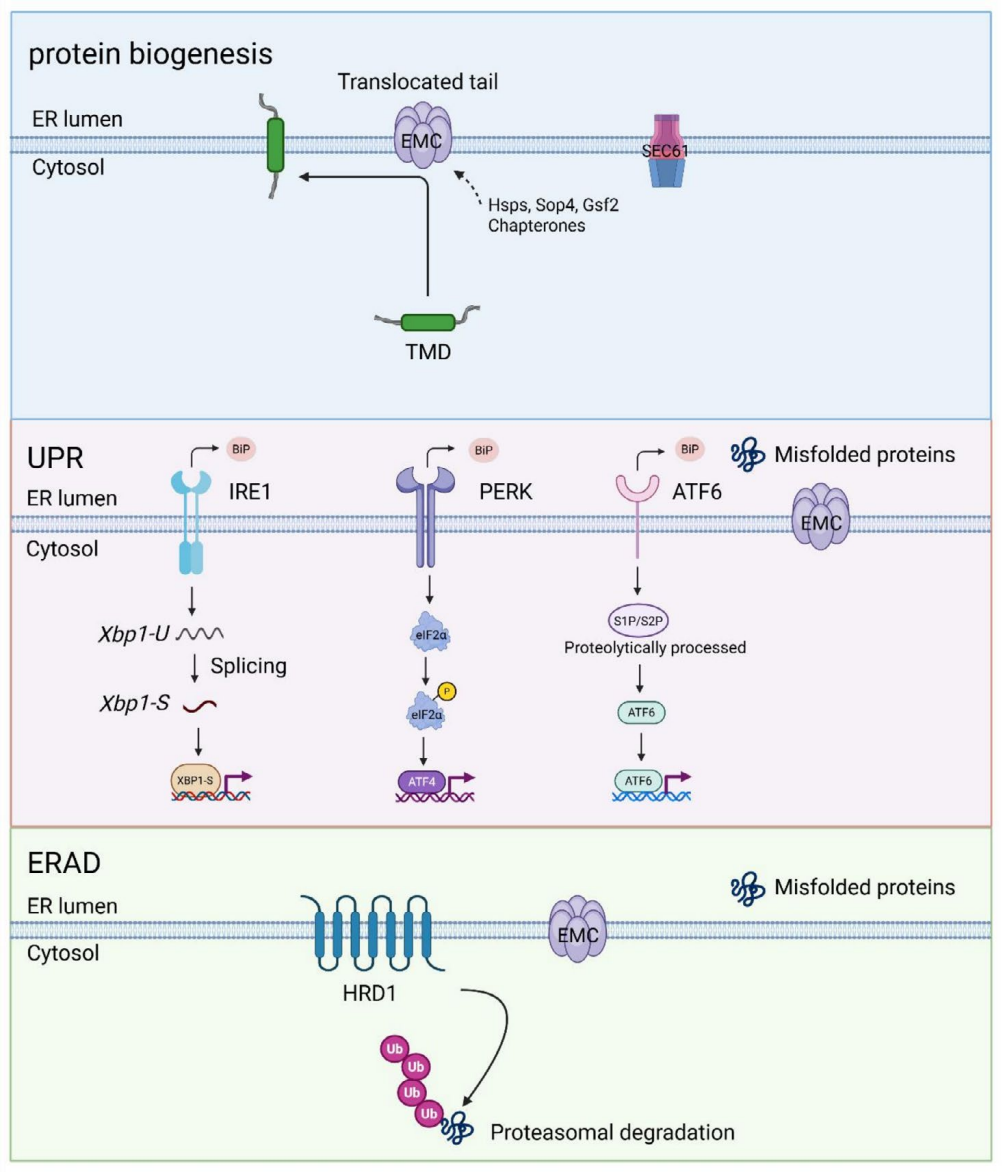
The core mechanisms of EMC function. The EMC facilitates the insertion and folding of TMDs with low hydrophobicity or multipass transmembrane proteins, a process that may involve substrate‐specific chaperones such as Sop4 and Gsf2. The UPR system comprises three sensors: IRE1α, PERK and ATF6. UPR activation inhibits global protein translation while enhancing the degradation of misfolded proteins. The ERAD system primarily functions through HRD1, which translocates misfolded proteins to the cytoplasm for degradation via the ubiquitin‐proteasome pathway.

When EMC3 is deficient or dysfunctional, hydrophobic client proteins, such as SP‐C and ABCA3, are retained in the ER lumen [8]. This results in the continuous accumulation of misfolded proteins, exceeding the cellular tolerance threshold and prompting EMC ‘molecular switch’ to forcibly transition from the ‘on’ state (folding assistance) to the ‘off’ state (stress compensation/degradation activation). In response, the cell alleviates ER stress through the coordinated action of multiple pathways, with specific mechanisms detailed below:

Tissue‐specific activation of UPR pathways in the lung: The UPR preferentially activates the IRE1α/XBP1 and PERK/eIF2α branches while specifically inhibiting the ATF6 branch. This selective pattern is specifically adapted for the lung to address folding crises of highly hydrophobic proteins. In response to the accumulation of unfolded proteins within the ER, the PERK pathway halts global protein synthesis by phosphorylating eIF2α, which reduces the influx of newly synthesized misfolded proteins. IRE1 is activated through a process involving BiP dissociation [66, 67, 68, 69], which facilitates IRE1 oligomerization, autophosphorylation and subsequent allosteric activation of the cytosolic RNase domain [66, 70]. IRE1‐dependent *XBP1* splicing (XBP1‐S) is predicted to be mediated through a process involving translational pausing during the ribosomal synthesis of XBP1 [71, 72, 73]. Together, these pathways establish the primary compensatory defence line (Figure 2).

The ERAD pathway is synergistically activated by XBP1‐S. Processed by the endonuclease activity of IRE1α, XBP1‐S acts as a key transcription factor that binds to promoters of core ERAD components (e.g., E3 ubiquitin ligase HRD1 and the retrotranslocation factor Derlin‐2) [74]. Subsequently, HRD1 catalyses K48‐linked polyubiquitination of the misfolded protein's cytosolic domain [75]. This ubiquitin tag serves as a recognition signal for the AAA+ ATPase p97 (also known as VCP or Cdc48 in yeast). VCP/P97 then extracts the ubiquitinated substrate from the membrane and unfolds it, delivering it to the 26S proteasome for degradation [76, 77, 78].

#### 3.2.3 The Molecular Trigger: Interaction With VCP/p97 and Beyond

Under basal conditions, EMC predominantly functions as a folding‐promoting TMD chaperone. In this state, it cooperates with substrate‐specific chaperones such as Sop4 and Gsf2 to maintain a stable conformation of its TMD‐binding cavity, thereby promoting the correct folding of client proteins [29, 79]. Additionally, EMC2 interacts with the cytosolic chaperone Hsp90, further integrating the complex into broader cellular proteostasis networks [80]. However, with persistent proteotoxic stress or alterations in its subunit composition, EMC undergoes a functional switch, transitioning from a folding facilitator to a component engaged in stress‐responsive pathways.

A key event in this switch may be the interaction between the cytosolic tail of EMC3 and the VCP/p97 [21, 22], likely reprogramming EMC from a folding assistant into a degradation‐facilitating factor. However, the structural and mechanistic basis of this transition remains unresolved. Importantly, it remains to be determined whether EMC engages VCP/p97 through direct recognition of substrate TMDs or whether the associated substrates are intrinsically misfolded in the absence of EMC. The widely held assumption that increased degradation of EMC clients equates to misfolding has yet to be experimentally validated.

In parallel, the EMC1 subunit can recruit DNAJC18 [81], whose cytosolic J‐domain engages an Hsp70–SGTA–Hsp105 complex at the ER membrane [82, 83, 84], providing an additional pathway for substrate handling under stress conditions.

### 3.3 EMC in the Lung: Spatiotemporal and Cell‐Specific Roles in Protein Homeostasis

The function of EMC in lung tissue is not uniformly conserved; rather, it exhibits dual specificity across both spatiotemporal (development versus adulthood) and cell type (epithelial versus mesenchymal) dimensions. This functional differentiation arises from variations in protein synthesis load and physiological demands at different developmental stages. Consequently, the lung provides a physiologically relevant system to dissect the diverse roles of EMC and to link molecular mechanisms to tissue‐level outcomes.

#### 3.3.1 Spatiotemporal Specificity: Shift in the Functional Necessity of EMC During Lung Development and Adulthood

As described above, during the late embryonic to neonatal period, EMC is essential for the synthesis and secretion of pulmonary surfactant by AT2 cells. A key feature of this stage is the function of EMC3 support for the folding and membrane integration of hydrophobic proteins such as SP‐C and ABCA3, which cannot be compensated by alternative mechanisms including the Sec61 translocon [52, 53]. *Emc3* deficiency in AT2 cells at this stage leads to immediate retention of client proteins in the ER lumen, forming massive misfolded protein aggregates that strongly activate the IRE1α/XBP1 and PERK/eIF2α branches of the PQC network, while specifically inhibiting ATF6 [8]. This triggers severe ER stress, disrupts surfactant homeostasis and ultimately impairs both embryonic development and neonatal pulmonary respiratory function.

In contrast, adult AT2 cells exhibit reduced dependence on EMC3. After birth, the cessation of burst surfactant synthesis leads to decreased protein synthesis demands, suggesting that EMC3 may primarily serve as a stress‐compensatory factor rather than a constitutive folding mediator [21]. However, the precise role of EMC3 in adult AT2 cells remains incompletely understood, highlighting an important area for further research.

#### 3.3.2 Cell Type Specificity: Contrasting Roles of EMC3 in Embryonic AT2 Cells and Lung Mesenchymal Cells

Distinct physiological functions among lung cell populations result in varying dependencies on EMC3. AT2 cells show the highest functional reliance, with EMC3 performing two core roles: (1) mediating the folding and membrane integration of hydrophobic proteins to support surfactant synthesis, and (2) participating in PQC to alleviate ER stress under challenging conditions [8].

By contrast, embryonic lung mesenchymal cells function to establish the interstitial scaffold of lung tissue through proliferation and differentiation, supporting epithelial cell growth, branching and maturation [85]. EMC3 deletion in embryonic lung mesenchymal cells leads to severe spindle assembly defects, continuous activation of cell cycle checkpoints and G2/M phase arrest. Since protein folding demand is low, classical UPR pathways are not induced; instead, cell death proceeds via apoptosis. Notably, similar effects are observed in human HeLa cells following EMC3 inhibition [22], indicating that this regulatory mechanism is conserved across species and cell types. These findings also provide insight into EMC's potential role in disorders characterised by abnormal proliferation, such as cancer.

Although in vivo studies on EMC in other tissues, such as intestinal epithelial cells, report that EMC deletion causes ER stress and impaired CFTR biogenesis [24], these studies do not exhibit the same spatiotemporal and cell type‐specific functional differentiation observed in the lung. The lung's structural complexity, cellular heterogeneity and postnatal functional transitions provide a conceptual framework for investigating tissue‐ and cell‐type‐specific roles of EMC complexes.

### 3.4 Integrative Perspectives and Future Directions

EMC function in pulmonary AT2 and mesenchymal cells integrates substrate‐specific folding, membrane insertion and quality control in a context‐dependent manner. The ‘molecular switch’ enables EMC to balance folding under homeostasis with degradation under stress, while cell type and developmental stage dictate the specific physiological demands on EMC activity.

The lung provides a powerful paradigm for dissecting EMC mechanisms in a tissue‐specific context. Future studies combining high‐resolution structural analyses, quantitative substrate dynamics and organotypic or in vivo models will be critical to resolve how subunit composition, conformational plasticity and cellular context collectively determine EMC function. Such integrative approaches are poised to advance our understanding of tissue‐specific proteostasis, stress adaptation and the contribution of EMC dysfunction to human disease.

## 4 From Paradigm to Pan‐Systemic Pathology—EMC Dysfunction Across Organs

Translating mechanistic insights of EMC into disease‐relevant contexts requires understanding both shared core mechanisms and tissue‐specific adaptations. The pulmonary model has established EMC's principles of substrate recognition, ‘molecular switch’ function and spatiotemporal/cell‐specific regulation. Extending these principles to other organ systems is critical for identifying pathogenic mechanisms and developing targeted therapeutic strategies. This section integrates clinical, genetic and experimental evidence (Table 2) to evaluate EMC dysfunction across neurological, retinal, cardiovascular, digestive, respiratory and oncological contexts.

**TABLE 2:** Summary of EMC‐related cancer diagnostic and prognostic markers.

Related EMC subunits	Significant protein expression difference	Significantly increase (+) or decrease (−)	Prognostic marker in	Favourable (+) or unfavourable (−)
EMC1	Lung SqCC	+	Colorectal cancer	+
	Colon AC	+	Liver cancer	−
	Pancreatic DAC	−	Renal cancer	+
EMC2	Renal cell carcinoma	−	Breast cancer	−
EMC3	Renal cancer	+	Renal cell carcinoma	−
EMC4	Renal cell carcinoma	−	Head and neck cancer	−
			Lung cancer	−
			Renal cancer	−
EMC5	Lung AC	+	Colorectal cancer	−
	lung SqCC	+		
	Colon AC	+	Renal cancer	+
	Renal cell carcinoma	−		
	Pancreatic DAC	−		
EMC6	Colon AC	+	Lung cancer	−
EMC7	Pancreatic DAC	−	Head and neck cancer	−
			Renal cancer	+
EMC8	Lung SqCC	+	Head and neck cancer	−
	Colon AC	+	Liver cancer	−
	Renal cell carcinoma	−	Renal cancer	+
EMC9	N/A	N/A	N/A	N/A
EMC10	Lung AC	+	Renal cancer	+
	Pancreatic DAC	−	Pancreatic cancer	+

*Note*: Data from The Human Protein Atlas (https://www.proteinatlas.org/).

Abbreviations: AC: Adenocarcinoma; DAC: Ductal Adenocarcinoma; SqCC: Squamous Cell Carcinoma.

### 4.1 Neurological and Retinal Diseases

**FIGURE 3:**
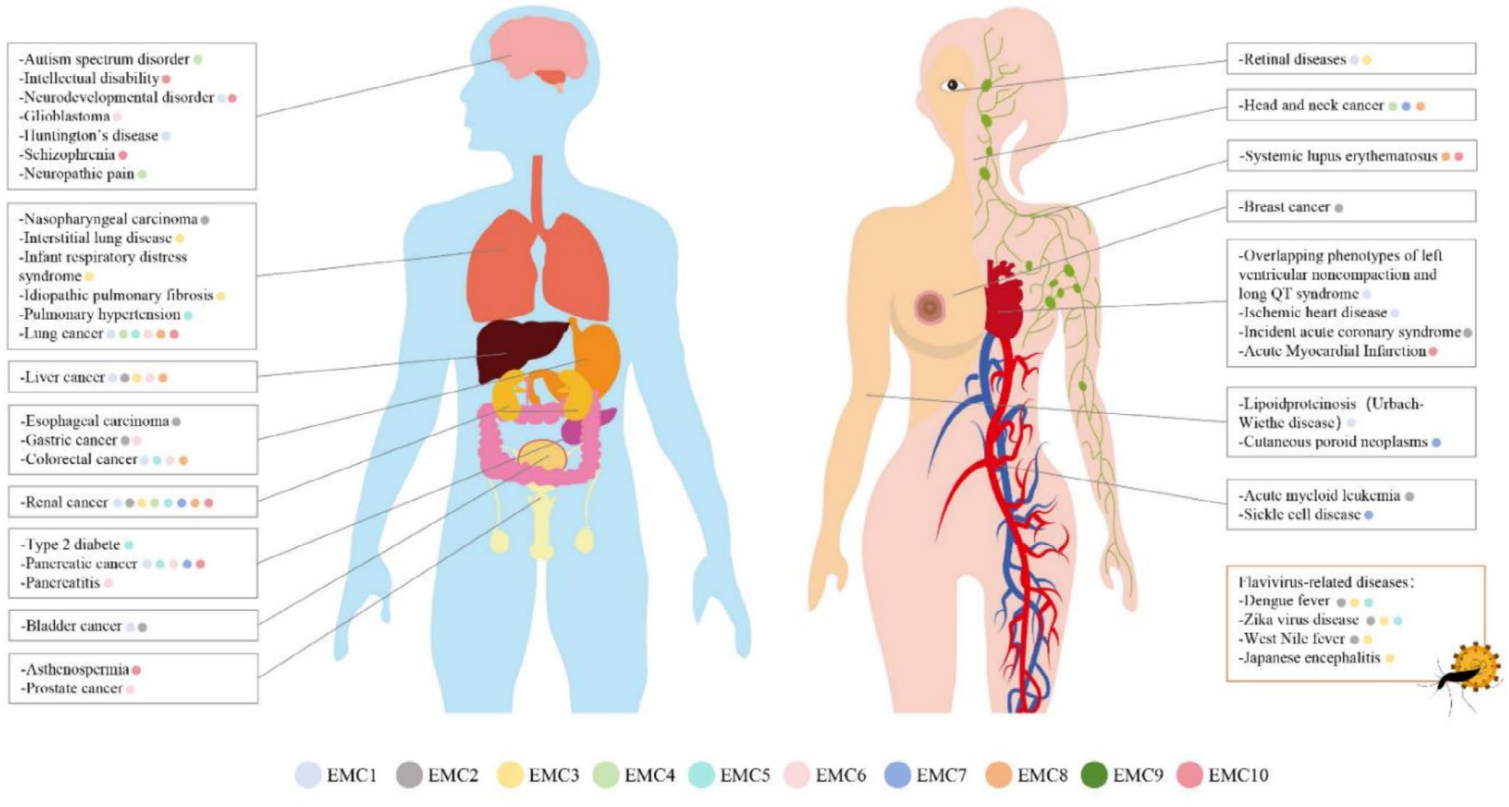
EMC subunits participate in diverse disease types. The subunits of the EMC complex are differentially involved in the pathogenesis, diagnosis and prognosis of diseases affecting nearly every tissue, organ and system of the human body. Notably, tumour tissues are frequently characterised by significant alterations in gene and protein expression levels of multiple EMC subunits.

EMC dysfunction in neurological and retinal tissue reflects both conserved molecular mechanisms and tissue‐specific adaptations (Figure 3). Similar to pulmonary AT2 cells, EMC acts as a ‘molecular switch’, balancing protein biogenesis and degradation. Loss of EMC3 in retinal cells disrupts the equilibrium, shifting the system from a homeostatic ‘ON’ state, supporting proper folding of client proteins, to a stress‐induced ‘OFF’ state that activates the UPR pathways, including the IRE1, PERK and ATF6 [17]. EMC3 is critical for M‐opsin stability, retinal precursor polarity and apoptosis regulation, mirroring cell type‐specific patterns observed in lung tissue [18] (Table 1).

Mechanistically, EMC subunits displayed both shared and unique roles across tissues. EMC1 and EMC3 contribute via distinct mechanisms: homozygous missense EMC1 variants (e.g., c.430G>A [p.Ala144Thr]) cause autosomal recessive retinal diseases [86], while EMC3 frameshift variants cause non‐syndromic retinitis pigmentosa. In the nervous system, EMC1 variants drive neurodevelopmental disorders [12, 87, 88, 89, 90, 91], characterised by global developmental delay, hypotonia, scoliosis and cerebellar atrophy. Structural analyses reveal that pathological EMC1 mutations (hEMC1^T82M^, hEMC1^G868R^) cluster in the hinge region between beta propellers where EMC7 binds, potentially disrupting complex stability [1]. Additionally, EMC1 likely facilitates FZD4 membrane localization [15] through luminal domain interactions, and pathogenic variants impair the Wnt signalling pathway and retinal vascular development. Beyond these core subunits, EMC10 variants are associated with intellectual disability and schizophrenia [92, 93, 94, 95, 96], while EMC4 dysregulation links to autism spectrum disorders and neuropathic pain [97].

These observations reinforce the concept that EMC‐mediated proteostasis and its ‘molecular switch’ function are conserved across cell types, but manifest differently depending on tissue‐specific client repertoires and developmental context. Resolving high‐resolution structures of disease‐associated EMC variants will clarify structure–function relationships, providing a mechanistic basis for molecular diagnosis and potential therapeutic targeting.

### 4.2 Respiratory Diseases

Infant Respiratory Distress Syndrome (IRDS) is a life‐threatening neonatal disorder characterised by alveolar collapse due to insufficient synthesis of pulmonary surfactant [98]. IRDS is primarily caused by pulmonary surfactant deficiency and clinically manifests as tachypnea, cyanosis and respiratory failure shortly after birth. Prematurity is a major risk factor; however, loss‐of‐function variants in EMC3 have been identified as a key pathogenic mechanism in a subset of hereditary IRDS, establishing EMC3 as a genetic contributor to this disease [8].

In postnatal and adult ILD, EMC3 function is critical in adult AT2 cells during the pathogenesis of ILD, particularly in the context of *SFTPC* ^*I73T*^ mutations [21]. In these cells, EMC3 mediates the ER‐to‐membrane transport and localization of the mutant proSP‐C(I73T). This aberrant trafficking causes the mutant protein to misaccumulate near the membrane, evade normal clearance mechanisms and induce cellular toxicity, thereby contributing to ILD pathology. Importantly, experimental deletion of EMC3 or inhibition of its interacting partner VCP/P97 mitigates the cytotoxic accumulation of mutant proSP‐C(I73T) and partially rescues the ILD phenotype, highlighting the functional relevance of EMC3 in mediating disease‐related protein mislocalization. While EMC3 normally supports proteostasis, in the context of pathogenic SFTPC variants, its transport function inadvertently facilitates mutant protein accumulation. Therefore, targeting the EMC3–VCP/P97 axis represents a promising strategy to alleviate mutant surfactant–induced lung injury, providing a direct mechanistic link between EMC function and potential therapeutic interventions.

Beyond its role in ILD, the broader implications of EMC dysfunction in respiratory diseases are poorly defined. The clinical association between EMC dysfunction and prevalent respiratory diseases, such as chronic obstructive pulmonary disease (COPD) and asthma, remains to be explored. Furthermore, the functions of EMC in pulmonary interstitial cells (e.g., fibroblasts) and immune cells (e.g., macrophages), which are crucial for fibrosis and inflammation [99, 100], remain largely unknown. Future research utilising complex models, such as lung organoids, is necessary to clarify the role of EMC in these additional cell types and disease contexts.

### 4.3 Cardiovascular Diseases

Dysfunctional EMC subunits are closely linked to the clinical presentation, progression and prognosis of cardiovascular diseases. Clinical genetic studies have found a strong association between pathogenic allelic variants of EMC1 and structural cardiac anomalies, with ischemic cardiomyopathy being the most prominent phenotype [9, 10, 11]. Affected patients commonly exhibit left ventricular hypertrophy, reduced systolic function and recurrent episodes of heart failure. EMC1 variants may lead to pleiotropic cardiovascular phenotypes that overlap [89]. Although the clinical association is well established, the precise molecular pathway by which EMC1 regulates myocardial proteostasis and influences cardiac development and function remains unknown. By analogy to the role of EMC3 in supporting the folding of SP‐C and ABCA3 in the lung, it is hypothesized that EMC1 may be indispensable for the biogenesis of cardiomyocyte‐specific membrane proteins, such as critical ion channels or receptors. Its dysfunction could, therefore, lead to the functional impairment of these proteins, resulting in structural cardiac abnormalities. Validating this hypothesis constitutes a crucial objective for future research.

Beyond monogenic disease risks imposed by rare variants, common genetic polymorphisms similarly implicate the EMC in the pathogenesis of diverse cardiovascular conditions. Genome‐wide association studies further implicate coding variants of EMC7 in modulating severity of vaso‐occlusive crises in sickle cell disease [101]. In contrast, EMC10 demonstrates a protective role, establishing a functional ‘counter‐regulatory’ pattern among EMC subunits. Preclinical and clinical data indicate that EMC10, secreted by cardiomyocytes and endothelial cells after myocardial infarction, may contribute to myocardial repair and vascular protection through paracrine mechanisms [102], This underscores the subunit‐specific and context‐dependent roles of EMC in cardiovascular homeostasis, reflecting both pathogenic and adaptive functions analogous to its dual roles in lung and other tissues, highlights the important role of the EMC lumen domain in cardiovascular diseases. This underscores the subunit‐specific and context‐dependent roles of EMC in cardiovascular homeostasis, reflecting both pathogenic and adaptive functions analogous to its dual roles in lung and other tissues, highlighting the important role of the EMC lumen domain in cardiovascular diseases.

### 4.4 Digestive Diseases

Building upon the principles elucidated in the lung—an organ featuring cells with high secretory demand and stringent reliance on EMC‐mediated membrane proteostasis, these principles extend to the digestive system. Organs of the digestive system, such as the liver and intestines, exhibit highly active secretory activities, requiring extensive synthesis, folding and trafficking of membrane and secretory proteins [103, 104, 105]. Simultaneously, these tissues encounter unique metabolic and stress‐related pressures. Under these challenging conditions, EMC subunits assume critical roles in maintaining protein homeostasis and organ function [24, 106, 107]. For example, EMC3 is vital for intestinal homeostasis by regulating the biogenesis of the CFTR, a polytopic membrane protein essential for ion and fluid transport [24] (Table 1). EMC3 deficiency leads to broad defects in polytopic membrane proteins, reduces mucus production by epithelial cells and triggers spontaneous intestine inflammation with heightened susceptibility to colitis [23], mirroring the dependence of pulmonary AT2 cells on EMC‐mediated biogenesis of surfactant proteins for alveolar homeostasis maintenance.

Beyond intestinal epithelial cells, EMC subunits also modulate hepatic metabolism. Loss of EMC10 exacerbates hepatic steatosis and activates the PERK‐eIF2α‐ATF4 signalling axis, whereas neutralisation of the secreted isoform scEMC10 ameliorates steatosis [106].

### 4.5 EMC in Cancer

The EMC complex plays a central role in modulating core cancer hallmarks, including metabolic reprogramming, evasion of cell death and maintenance of stemness, primarily through stabilising oncogenic client proteins. Its functions are highly context‐dependent, displaying both tumour‐suppressive and oncogenic roles depending on cell type and microenvironment. For instance, EMC3 promotes proliferation, migration, invasion and metastasis in hepatocellular carcinoma (HCC), with high expression correlating with poor prognosis [108, 109, 110]. Conversely, EMC3 knockdown in HeLa cells induces cell cycle defects and apoptosis, consistent with its role in mitotic spindle assembly in lung mesenchymal cells [21], highlighting its essential function in cell cycle progression and survival in specific contexts and connecting EMC dysfunction to tumorigenic mechanisms.

Other EMC subunits also show distinct and context‐specific roles in tumorigenesis. EMC2 promotes ferroptosis resistance in triple‐negative breast cancer by stabilising a key enzyme in cholesterol biosynthesis [111], and has been identified as a critical ferroptosis‐related gene in HCC and oesophageal cancer [112, 113]. EMC6 exhibits opposing roles: it suppresses glioblastoma via autophagy activation but acts as an oncogene in lung adenocarcinoma [114]. Recent study unveiling EMC6 as a novel pathogenic determinant in HCC, diminished expression level of EMC6 was associated with poor prognosis of HCC patients [115]. EMC1 demonstrates high mutation frequencies in multiple cancers, contributing to drug resistance and the maintenance of cancer cells [108, 116].

EMC subunits function not only as regulators of tumorigenesis but also as potential clinical indicators for survival prognosis assessment (partial data are summarised in Table 2). Clinical and multi‐omics analyses further underscore the translational relevance of EMC subunits. Expression levels of EMC1, EMC4, EMC5, EMC6, EMC8 and EMC10 correlate with prognosis in lung cancer (Table 2, Human Protein Atlas database), while EMC4, EMC7 and EMC8 show biomarker potential in head and neck cancer [117]. EMC8 has been shown to be associated with poor prognosis and insufficient CD8^+^ T cell infiltration in head and neck squamous cell carcinoma [117]. In HCC, multi‐omics studies reveal that core EMC subunits (EMC1‐3, EMC6 and EMC10) exhibit alterations strongly linked to patient survival [108, 112, 118]. EMC9, though less well studied, is upregulated in retinal malignancies and promotes oncogenesis via circEMC9‐mediated PI3K/AKT activation [119], suggesting broad pro‐tumorigenic roles across various cancer contexts.

The context‐dependent duality of EMC function in cancer reflects tissue‐specific client repertoires, tumour microenvironment‐driven reprogramming and differential dependency of oncogenic signalling pathways on EMC clients. Future research should prioritise systematic mapping of EMC ‘client proteome’ across tumour types and dissecting how EMC conformational states respond to microenvironmental cues. Such studies will inform whether targeting EMC functions can overcome therapy resistance driven by aberrant membrane protein stability, linking mechanistic insights from pulmonary and other tissue models to translational cancer strategies.

### 4.6 EMC Across Organs: Conserved and Tissue‐Specific Roles

EMC dysfunction disrupts core processes of membrane protein biogenesis and proteostasis across multiple tissues, reflecting both conserved mechanisms and tissue‐specific adaptations. In the nervous and retinal systems, EMC1 and EMC3 variants impair folding or localization of key client proteins (e.g., FZD4, M‐opsin), paralleling the cell type‐specific regulatory patterns seen in pulmonary AT2 cells [15, 18]. In cardiovascular tissues, EMC1 and EMC2 variants compromise cardiomyocyte and endothelial protein biogenesis, whereas EMC10 exhibits protective paracrine functions post‐injury. In the digestive system, EMC3 supports CFTR biogenesis and secretory homeostasis, while EMC10 regulates hepatic metabolism, illustrating conserved roles in highly secretory organs analogous to the lung [24, 89, 120]. In cancer, EMC subunits exert context‐dependent effects on proliferation, apoptosis and metabolic reprogramming, emphasising client and microenvironment‐specific regulation [109, 114].

These findings highlight the potential of EMC‐targeted strategies across diseases. Systematic mapping of EMC client repertoires and the functional impact of disease‐associated variants will be critical to understand structure–function relationships, proteostasis network vulnerabilities and tissue‐specific dependencies. Building on mechanistic insights from the lung, such integrative approaches can inform precision interventions aimed at restoring proteostasis or modulating EMC pathways in neurological, cardiovascular, digestive and oncological disorders, laying the foundation for the therapeutic strategies discussed in the next chapter.

## 5 Therapeutic Potential of EMC: Promise and Unmet Challenges

Significant advances have been made in understanding the roles of EMC in membrane protein biogenesis [19, 52], protein quality control and disease associations. However, critical gaps persist regarding the fundamental mechanisms, tissue‐specific regulation and the translation of findings into clinical applications.

### 5.1 From Structure to Mechanism

The structural basis of substrate selectivity in EMC remains unresolved [42, 44]. Specifically, the interaction patterns between the TMDs of tissue‐ or disease‐specific substrates and EMC's hydrophilic cavity and positively charged regions remain unclear [50]. To tackle this challenge, future research should prioritise the determination of dynamic complex structures of the EMC bound to tissue‐specific substrates through high‐resolution cryo‐electron microscopy (cryo‐EM). This methodology will elucidate key interaction sites and hydrophobic interactions between substrate TMDs and the EMC's hydrophilic cavity and positively charged areas. Establishing a quantitative model that correlates substrate physicochemical properties with EMC binding efficiency will fundamentally clarify the structural basis for EMC substrate selectivity.

There exists a notable gap in understanding the organ‐specific mechanisms of EMC. In critical tissues like the cardiovascular system, the physiological functions of key EMC domains (e.g., the luminal domains of EMC3, EMC4 and EMC6) have yet to be validated. To address this gap, future research should prioritise cardiovascular models, employing CRISPR‐Cas9 to engineer subunit‐specific EMC point mutations [121]. Subsequent integration of co‐immunoprecipitation‐mass spectrometry (CoIP‐MS) with functional assays in these models would enable a direct test of the hypothesis that EMC luminal domains are required for the membrane integration of cardiomyocyte‐specific client proteins.

### 5.2 Systematically Validating the Spatiotemporal and Cell‐Type Specificity of EMC Across Tissues

While this review synthesises evidence for EMC functions, a unified picture of the field must also reconcile inconsistent findings. A key example is in the cell‐type‐specific UPR response to EMC depletion. As detailed in Section 3, loss of EMC3 in pulmonary AT2 cells induces a specific UPR signature involving the IRE1α/XBP1 and PERK/eIF2α branches [8], whereas neuronal EMC dysfunction activates all three UPR pathways [13]. These divergent outcomes reflect the cell‐type‐specific clientome and proteostasis network capacity that shapes EMC function.

Inconsistencies extend beyond UPR signalling. Moreover, the primary disease‐associated EMC subunit varies with context: EMC3 is a key etiological factor in lung and intestinal pathologies [8, 24], contrasting with EMC1 and EMC10 in specific neurological and cardiovascular disorders [102, 114]. Similarly, the fundamental role of EMC is contested, being viewed as either a broad insertase or a specialised chaperone for problematic membrane proteins.

The varying phenotypic outcomes likely reflect differences in developmental stage, cellular metabolic state and the presence of compensatory pathways. Crucially, the precise mapping of these variables remains elusive. To address these gaps, we propose an integrated strategy utilising single‐cell spatiotemporal multi‐omics technologies, such as spatial transcriptomics and proteomics, to systematically map the expression landscape of EMC across various human tissues, including both high‐ and low‐secretion organs throughout the lifespan. This approach aims to identify the spatiotemporal expression patterns of EMC in high‐secretion tissues, such as the lung and intestine, providing direct evidence for the hypothesis of a ‘developmental‐to‐adult’ functional transition. It will also reveal the differences in the regulatory networks of EMC in low‐secretion or specialised tissues, such as neural and cardiovascular tissues, elucidating the basis for its tissue‐specific functions. Additionally, this strategy will decipher the functional heterogeneity of EMC across different cell types within the same organ at single‐cell resolution, systematically clarifying its cell‐type‐specific mechanisms. Through these investigations, this research has the potential to establish a comprehensive functional framework for EMC across tissues, throughout developmental stages and at the cellular level, laying a solid foundation for understanding its role in various physiological and pathological conditions.

### 5.3 Therapeutic Strategies and Perspectives for Clinical Translation

The EMC has emerged as a potential therapeutic target for various related diseases. However, its broad and complex physiological functions present significant challenges for clinical translation. Current therapeutic strategy development must prioritize three core areas: overcoming systemic toxicity, enhancing targeting specificity and exploring innovative intervention dimensions. This effort primarily follows three main approaches: directly targeting EMC subunits, targeting EMC‐substrate interactions and precision interventions based on isoform‐specific functional differentiation. The ultimate goal is to achieve a balance between therapeutic efficacy and safety.

#### 5.3.1 Direct Targeting of EMC Subunits

The development of small‐molecule inhibitors targeting EMC subunits is still in the early exploratory stages, with no clinically validated drug candidates available to date. Given the central role of EMC in the biosynthesis of various membrane proteins, systemic inhibition may disrupt critical physiological functions in multiple organs. For example, targeting EMC3 for the treatment of ILD may simultaneously impair intestinal epithelial homeostasis. Future efforts should focus on advancing tissue‐targeted delivery technologies to enable precise targeting of EMC subunit inhibitors. For example, in treating ILD, lung‐targeted small‐molecule inhibitors of EMC3 could be developed by modifying the lipophilicity of drug molecules, conjugating them with lung epithelial cell‐specific ligands (such as SP‐C receptors on AT2 cells) [122] or using inhalable nanocarrier delivery systems. This would preferentially enrich the drugs in lung lesions while minimizing impacts on normal organs, including the intestines [123]. Additionally, CRISPR screening technology can be utilized to identify tissue‐specific interacting proteins of EMC subunits, enabling the design of inhibitors that only block EMC function in diseased tissues and reduce off‐target toxicity [124].

#### 5.3.2 Targeting EMC‐Substrate Interactions

Compared to direct inhibition of EMC subunits, the development of small‐molecule modulators that selectively target the interactions between EMC and disease‐related substrates has emerged as a more feasible and precise therapeutic strategy, primarily because it can avoid systemic organ toxicity by preserving normal EMC‐substrate interactions. This approach has been initially supported by research findings. When iAT2 cell models derived from ILD patients with SFTPC^*I73T*^ mutation were treated with the VCP/P97 inhibitor CB5083, the ball‐like structures formed by mutant cells were restored to the monolayered alveolosphere morphology characteristic of normal iAT2 cells. This treatment also effectively inhibited the misprocessing and mistrafficking of proS‐PC (I73T) [21].

#### 5.3.3 Isoform‐Specific Targeting: Novel Therapeutic Opportunities Enabled by Isoform Functional Differentiation

Distinct isoforms of EMC subunits offer novel targets for precise therapeutic intervention. For instance, the secreted isoform EMC10 (scEMC10) has been identified as a key driver of obesity, insulin resistance and hepatic steatosis [106, 125]. Preclinical studies demonstrate that its neutralising antibody can effectively ameliorate these metabolic dysfunctions [126]. However, current understanding remains insufficient regarding the differential expression and functional roles of EMC splice variants under disease conditions. In the future, it is necessary to clarify the expression differences of EMC splice variants between diseased and normal tissues through technologies such as transcriptome sequencing and analyse their tissue‐ or disease‐specific functions [127]. Based on this knowledge, intervention molecules targeting the interaction interface between EMC and disease substrates or disease‐specific subtypes should be developed. Meanwhile, single‐cell sequencing, proteomics and other technologies should be combined to map the essential substrate profile of EMC in different tissues [51], so as to provide a basis for accurately avoiding toxicity risks.

## Author Contributions

X.T., Q.M. and X.L. designed the manuscript. Y.Q., Y.S. and Y.H. drafted the manuscript. X.T. and W.W. revised the manuscript. All authors contributed to the article and approved the final version for submission.

## Funding

This work was supported by National Key Research and Development Program of China, 2022YFA0806200. Guangdong Basic and Applied Basic Research Foundation, 2023A1515010279, 2023A1515110474.

## Conflicts of Interest

The authors declare no conflicts of interest.

## Data Availability

Data sharing is not applicable to this article as no new data were created or analysed in this study.
